# Regimen

**Published:** 1856-10

**Authors:** 


*Regimen.*—Dr. James Jackson, in his letters to a young physician, advocates an exclusive vegetable diet, both as a remedy and a preventive measure in epilepsy and apoplexy. Although patients may rebel against the prescription, if made to embrace the remainder of their lives, they will generally become reconciled to it if recommended temporarily, so as to become more indifferent on the subject than they had anticipated. Exercise is enjoined, mental perturbation disapproved, and the patient advertised not to return to animal food so long as he has very good health without it. In phthisis and hemoptisis on the contrary, he recommends animal food, milk, and a farineous diet, to which should be added fruit, and other articles of a laxative character, in case of a tendency to habitual constipation. Exercise in the open air, he considers of all things the most important in these diseases, which should be carried as far as the vigor of the patient will permit. It should not be done rashly, but boldly. The great object is, to prevent the cachexy, if it has not appeared, or to overcome it when it has, by such measures as will tend to increase the general vigor of the system, trusting to the natural efforts to overcome the disease. With the body properly protected by suitable clothing the patient is advised to live pretty much out of doors. For the relief of hemoytysis he recommends a combination of sulphate of copper and opium. In an urgent case he gave one grain each of these remedies, and repeated the dose in twelve hours. During fifty years practice he had only met with two cases in which this hemorrhage proved fatal in phthisis.—*Med. Chronicle.*

